# Ultrafast Umklapp-assisted electron-phonon cooling in magic-angle twisted bilayer graphene

**DOI:** 10.1126/sciadv.adj1361

**Published:** 2024-02-09

**Authors:** Jake Dudley Mehew, Rafael Luque Merino, Hiroaki Ishizuka, Alexander Block, Jaime Díez Mérida, Andrés Díez Carlón, Kenji Watanabe, Takashi Taniguchi, Leonid S. Levitov, Dmitri K. Efetov, Klaas-Jan Tielrooij

**Affiliations:** ^1^ Catalan Institute of Nanoscience and Nanotechnology (ICN2), BIST and CSIC, Campus UAB, 08193 Bellaterra (Barcelona), Spain.; ^2^ ICFO - Institut de Ciencies Fotoniques, The Barcelona Institute of Science and Technology (BIST), Castelldefels 08860, Spain.; ^3^ Fakultät für Physik, Ludwig-Maximilians-Universität, Schellingstrasse 4, München 80799, Germany.; ^4^ Munich Center for Quantum Science and Technology (MCQST), München, Germany.; ^5^ Department of Physics, Tokyo Institute of Technology, Tokyo, Japan.; ^6^ Research Center for Functional Materials, National Institute for Material Sciences, Tsukuba, Japan.; ^7^ International Center for Materials Nanoarchitectonics, National Institute for Material Sciences, Tsukuba, Japan.; ^8^ Department of Physics, Massachusetts Institute of Technology, Cambridge, 02139 MA, USA.; ^9^ Department of Applied Physics, TU Eindhoven, Den Dolech 2, Eindhoven 5612 AZ, Netherlands.

## Abstract

Understanding electron-phonon interactions is fundamentally important and has crucial implications for device applications. However, in twisted bilayer graphene near the magic angle, this understanding is currently lacking. Here, we study electron-phonon coupling using time- and frequency-resolved photovoltage measurements as direct and complementary probes of phonon-mediated hot-electron cooling. We find a remarkable speedup in cooling of twisted bilayer graphene near the magic angle: The cooling time is a few picoseconds from room temperature down to 5 kelvin, whereas in pristine bilayer graphene, cooling to phonons becomes much slower for lower temperatures. Our experimental and theoretical analysis indicates that this ultrafast cooling is a combined effect of superlattice formation with low-energy moiré phonons, spatially compressed electronic Wannier orbitals, and a reduced superlattice Brillouin zone. This enables efficient electron-phonon Umklapp scattering that overcomes electron-phonon momentum mismatch. These results establish twist angle as an effective way to control energy relaxation and electronic heat flow.

## Teaser

Electron cooling in moiré graphene is ultrafast even at cryogenic temperatures due to electron-phonon Umklapp scattering.

## INTRODUCTION

Moiré superlattices based on layered materials are a recently developed material platform in which twist angle controls the effective lattice constant. As the twist angle decreases, the larger moiré unit cell corresponds to a smaller electron momentum. This tunes the relative strength of the kinetic energy of electrons and the interaction energy between them. In magic-angle twisted bilayer graphene (MATBG), these interactions result in a rich phase diagram that includes superconductors (*1*–*4*), correlated insulators (*5*), and orbital magnets (*2*, *6*). In transition metal dichalcogenides, correlated insulating (*7*, *8*) and ferromagnetic states (*9*) are observed over a broad range of angles, with moiré excitons (*10*, *11*) providing a testbed for exploring Hubbard model physics and quantum computation (*8*, *12*). The technological implications of moiré materials are substantial, with applications envisaged in superconducting circuits (*13*–*15*), energy harvesting (*16*), nonlinear optics (*17*, *18*), and optical sensing (*19*–*21*).

The moiré potential also modifies the phonon spectra for small twist angles (*22*). This results in phonon renormalization in MoS_2_ homobilayers (*23*) and the emergence of phonon minibands in twisted bilayer graphene (*24*). Theoretical studies predict that the moiré potential strongly affects electron-phonon coupling (*25*–*28*), which has important implications for electrical transport, excited-state relaxation dynamics, and beyond.

Excited-state relaxation measurements are particularly well-suited probes to quantitatively assess electron-phonon coupling. The relaxation dynamics in graphene after excitation involve thermalization of high-energy carriers through carrier-carrier scattering within tens of femtoseconds (*29*), creating a hot carrier distribution that subsequently cools via electron-phonon interactions. Inelastic electron-phonon scattering allows electrons to gain (lose) energy by the absorption (emission) of a phonon. In graphene, cooling typically occurs via the emission of optical and acoustic graphene phonons and near-field coupling to substrate phonons (*30*–*40*). In all cases, cooling becomes increasingly slow for lower lattice temperatures.

Experimental studies of the relaxation dynamics of twisted bilayer graphene have so far been limited to large twist angles (θ > 5°). In these systems, a dark exciton state emerges between van Hove singularities, leading to slower dynamics (*41*, *42*). At θ = 30°, twisted bilayer graphene forms a quasi-crystal, which allows for Umklapp electron-electron scattering that couples the two graphene layers (*43*, *44*). Recent Raman spectroscopy measurements suggest an enhanced electron-phonon coupling strength for twist angles above 0° and below 3° (*45*). However, direct experimental measurements of moiré-enhanced electron-phonon coupling and its implications for cooling dynamics and technological applications are lacking, nor is there any clear experimental evidence that explains the origin of the enhanced coupling.

**Fig. 1.**
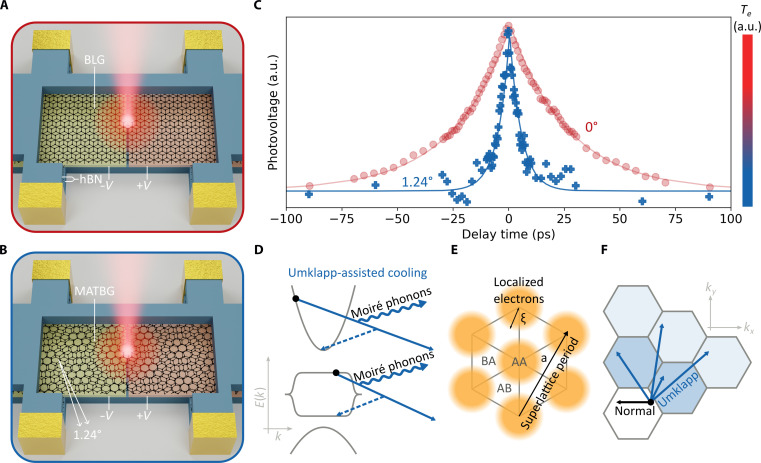
Excited carrier relaxation in MATBG. (**A** and **B**) Illustration of the hBN-encapsulated BLG device with 0° twist angle (A) and the hBN-encapsulated MATBG device with small twist angle (B), each equipped with split gates. By applying voltages of opposite sign (±*V*) to the split gates, we create a pn-junction (the interface between yellow and orange regions). Illuminating the junction generates a photovoltage via the photothermoelectric effect, which is proportional to the electron temperature (*T_e_*). We obtain the temperature dynamics either by using two ultrashort laser pulses separated in time by a variable temporal delay (*29*, *46*, *47*) or by using two spectrally narrow laser beams with variable frequency detuning (*48*, *49*). (**C**) Photovoltage as a function of time delay for a lattice temperature of 25 K. The decay, which represents the cooling dynamics, is much faster in MATBG (blue pluses) than BLG (red circles). a.u., arbitrary units. (**D**) Schematic of the MATBG band structure. Umklapp scattering processes (solid arrow) allow for efficient electron (black circle) relaxation via coupling to moiré phonons (wiggly lines). These Umklapp processes can occur in both the flat and the dispersive bands. The dashed arrows represent the equivalent final state in the first Brillouin zone. (**E**) Schematic of the compressed Wannier orbitals of radius ξ. Electrons are localized to AA sites in the reconstructed superlattice. (**F**) Umklapp scattering processes (blue arrows) couple electrons in the first Brillouin zone (white hexagon) to large-momentum phonons in higher-order Brillouin zones (blue hexagons).

Here, we report the observation of ultrafast cooling in MATBG and attribute this to the occurrence of Umklapp-assisted electron-phonon scattering. We directly probe the electron-phonon interaction by measuring carrier cooling dynamics using two well-established optoelectronic techniques: time-resolved photovoltage microscopy (TrPV) (*29*, *46*, *47*) and continuous-wave photomixing (*48*, *49*). We make a direct comparison between a nontwisted Bernal bilayer graphene (BLG) sample (θ = 0°; see Fig. 1A) and two near-MATBG samples (with twist angles of 1.24° and 1.06°; see Fig. 1B). This is an ideal comparison because only the twist angle varies between the three devices. At low temperature, the cooling dynamics are much faster in MATBG than in nontwisted bilayer graphene (see Fig. 1C). This result highlights the crucial role of the moiré pattern and suggests the emergence of an enhanced electron-phonon interaction in small twist angle systems. We explain the observed relaxation dynamics using a theoretical model based on Umklapp-assisted electron-phonon scattering, which can occur in both the dispersive and flat bands of MATBG (see Fig. 1D). The Umklapp processes are enabled by the presence of compressed electronic Wannier orbitals (see Fig. 1E) and the superlattice with reduced Brillouin zone (see Fig. 1F). Whereas phonon-phonon Umklapp scattering is a ubiquitous process that typically governs the thermal conductivity of semiconductors and insulators and electron-electron Umklapp scattering has been observed in some cases, c.f. (*43*, *44*, *50*, *51*), this is not the case for electron-phonon Umklapp scattering.

## RESULTS

We study relaxation dynamics in MATBG and BLG Hall bar devices as shown in Fig. 1 (A and B) (see Materials and Methods for details on the device fabrication and characterization and figs. S1 to S3). In both devices, the graphene is encapsulated by hexagonal BN (hBN). These devices enable both electrical and optoelectronic measurements, as they are equipped with a split gate (lateral separation, 300 nm) that we use to create a photoactive pn-junction region. The resistance map as a function of the gate voltage applied to each of the two sides of the split gate, shown in fig. S4, displays clear peaks at the usual Dirac points with vanishing carrier density. The MATBG device exhibits additional peaks at integer fillings of the superlattice unit cell. By illuminating the pn-junction with light, a photovoltage is generated via the photothermoelectric effect. This effect has a characteristic sixfold symmetry in dual-gate photovoltage maps, as shown in fig. S5 for both devices. This indicates that the measured photovoltage is a direct probe of the electron temperature (*52*). This electron temperature is established via electron-electron interactions that take place on a timescale of tens of femtoseconds for nontwisted bilayer graphene (*53*). The electron-electron interactions are even stronger in twisted bilayer graphene, leading to even faster carrier thermalization rates (*54*).

We study hot electron cooling using ultrafast TrPV as implemented in (*29*, *36*) and continuous-wave heterodyne photomixing (CW-PM) as implemented in (*49*). In the former, ultrashort laser pulses with a wavelength of 1030 nm are incident upon the pn-junction, whereas for the latter two, continuous-wave lasers with a wavelength around 1550 nm are used. In both cases, we probe the generated photovoltage. These two techniques allow us to obtain directly the carrier cooling dynamics—in the time domain by varying the time delay between two ultrashort laser pulses and in the frequency domain by varying the spectral detuning of two spectrally narrow laser beams. We combine these two techniques to probe the dynamics in different electron temperature regimes: The time-resolved measurements use ultrashort pulses, leading to higher peak electron temperatures than the frequency-resolved measurements that use continuous-wave light. In both cases, we use relatively low incident fluences (typically 60 μJ cm^−2^) to avoid that the electron temperature dominates the observed dynamics, instead of the lattice temperature.

**Fig. 2.**
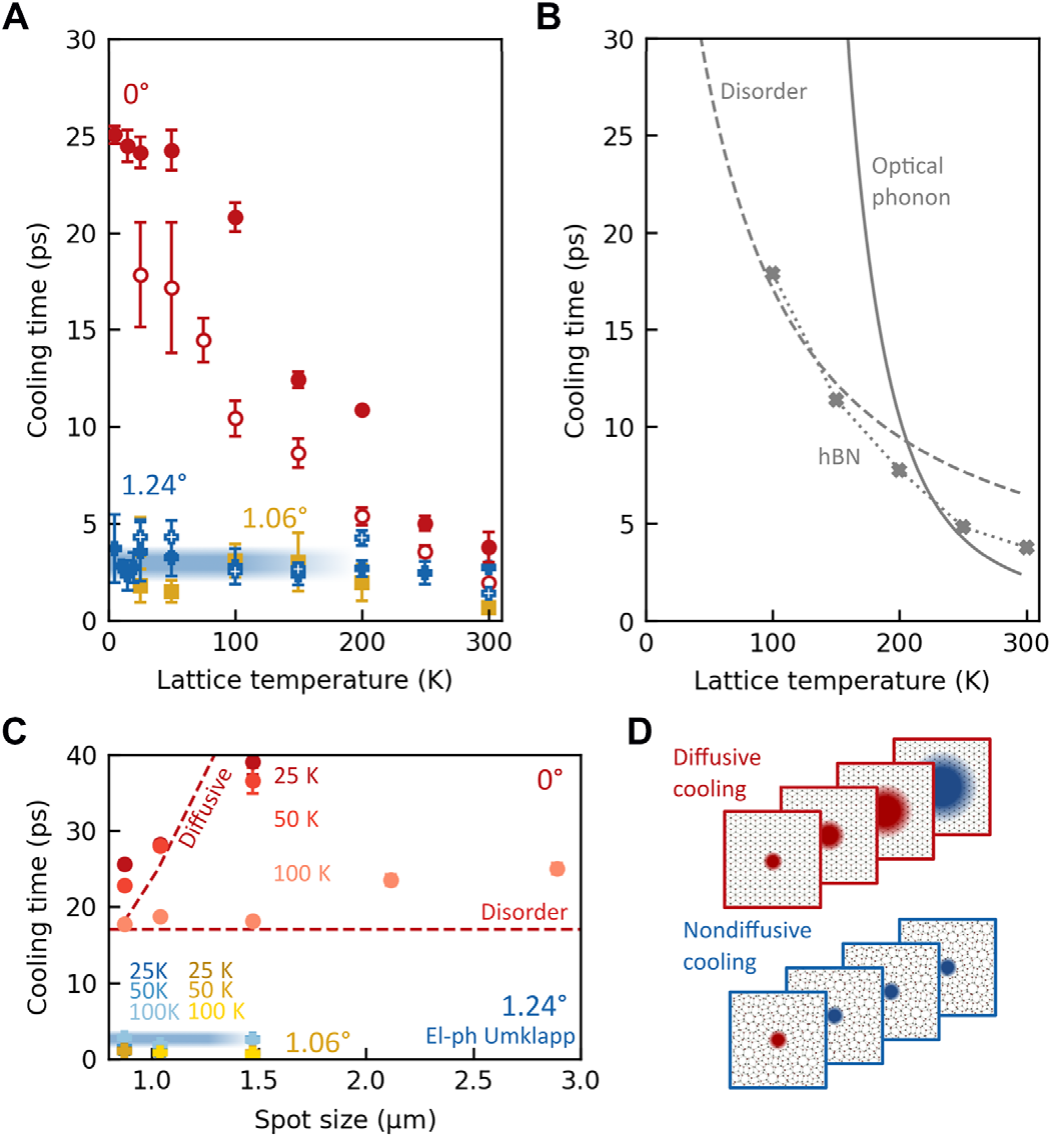
Relaxation mechanisms in MATBG and BLG. (**A**) Cooling time as a function of lattice temperature. In MATBG (1.24°, blue pluses; 1.06°, yellow squares), the cooling time is constant between 5 and 300 K (3 ps; blue line). For BLG (0°, red circles), it is greater at lower temperatures. The filled (open) symbols correspond to TrPV (CW-PM) measurements. Error bars represent the statistical spread across different gate voltages. (**B**) Calculated cooling times due to optical phonon emission based on (*55*), hBN hyperbolic phonon scattering based on (*36*, *37*), and disorder-assisted scattering based on (*31*, *33*, *34*). For all mechanisms, cooling is slower at lower temperatures. (**C**) Laser spot size dependence of the cooling time. The strong dependence in BLG at 25 and 50 K is a signature of diffusive cooling. This effect is weaker at 100 K, where disorder-assisted cooling becomes substantial. The effect is absent in MATBG for these spot sizes. The thick blue line in (A) and (C) represents the cooling time obtained from the low-temperature model of Umklapp-assisted cooling (see main text). (**D**) Schematics of diffusive cooling for BLG (top) and its absence for MATBG (bottom).

Both techniques independently show that charge carriers cool much faster in both MATBG samples than in BLG at low temperature (see Fig. 2A and figs. S6 to S9). In BLG, the cooling time increases from 3 to 25 ps as the temperature decreases from 300 to 5 K, which is expected as it takes longer for hot carriers to couple to phonons at lower temperature due to the reduced phonon occupation (*30*, *31*). Notably, in MATBG, the cooling time remains short, around 3 ps, across a broad temperature range (5 to 300 K). This suggests the involvement of low-energy phonons that still have occupation at such low temperature, which are likely phonons originating from the superlattice. The moiré potential breaks the original linear phonon dispersion into minibands with enhanced density of states (DOS) (*24*). The energy of the lowest band is below 1 meV corresponding to temperatures below 10 K.

To understand the origin of the observed cooling dynamics, we first consider the case of relaxation through energy transfer to phonons in nontwisted BLG (see Fig. 2B). Coupling to optical phonons is highly inefficient at low temperature due to the large optical phonon energy, which is >160 meV, corresponding to *T* > 2000 K (*39*, *55*). Coupling between electrons and acoustic phonons is normally also inefficient because of the reduced phase space available for scattering and would give cooling times well above a nanosecond below 25 K (*30*). The presence of defects can help overcome the electron-phonon momentum mismatch through disorder-assisted cooling, which speeds up this acoustic phonon cooling process (*31*, *33*–*35*). However, even with this mechanism, we expect cooling times between 0.1 and 10 ns for the lowest temperatures, depending on the electron mean free path (see Materials and Methods). Cooling can also occur via near-field radiation to substrate modes, which are usually phonons. The hyperbolic phonon modes present in hBN are particularly efficient heat sinks (*36*, *37*). However, this cooling channel becomes less efficient at lower lattice temperatures as well: The cooling time is around 10 ps for a lattice temperature of 200 K, while getting increasingly slow at lower temperatures.

Since all currently known cooling mechanisms that involve phonon emission show increasingly slow cooling at lower lattice temperatures, we consider diffusive cooling. Here, electronic heat diffuses out of the initially excited hotspot, thus leading to a lower average electron temperature (*38*, *56*). In this diffusive cooling mechanism, the cooling time will thus depend on laser spot size. For nontwisted BLG, we observe an increase in cooling time for larger spot sizes, which is the largest for the lowest temperatures (25 and 50 K) (see Fig. 2, C and D). At higher temperatures, the cooling length is shorter (*56*), and therefore diffusive cooling has a smaller contribution. We thus understand the cooling dynamics for nontwisted BLG from a combination of disorder-assisted and diffusive cooling. Our calculations of the cooling time based on these two mechanisms are close to the experimentally observed ones (see Materials and Methods for details on the calculations). For MATBG, we observe no dependence of the cooling time on spot size (see Fig. 2C), which suggests that diffusive cooling does not play a role for this system. It furthermore shows that the cooling time is insensitive to small variations in twist angle (δθ ≈ ±0.04°; see fig. S3). This means that in twisted bilayer graphene, electron heat stays localized, rather than spreading in space (see Fig. 2D).

**Fig. 3.**
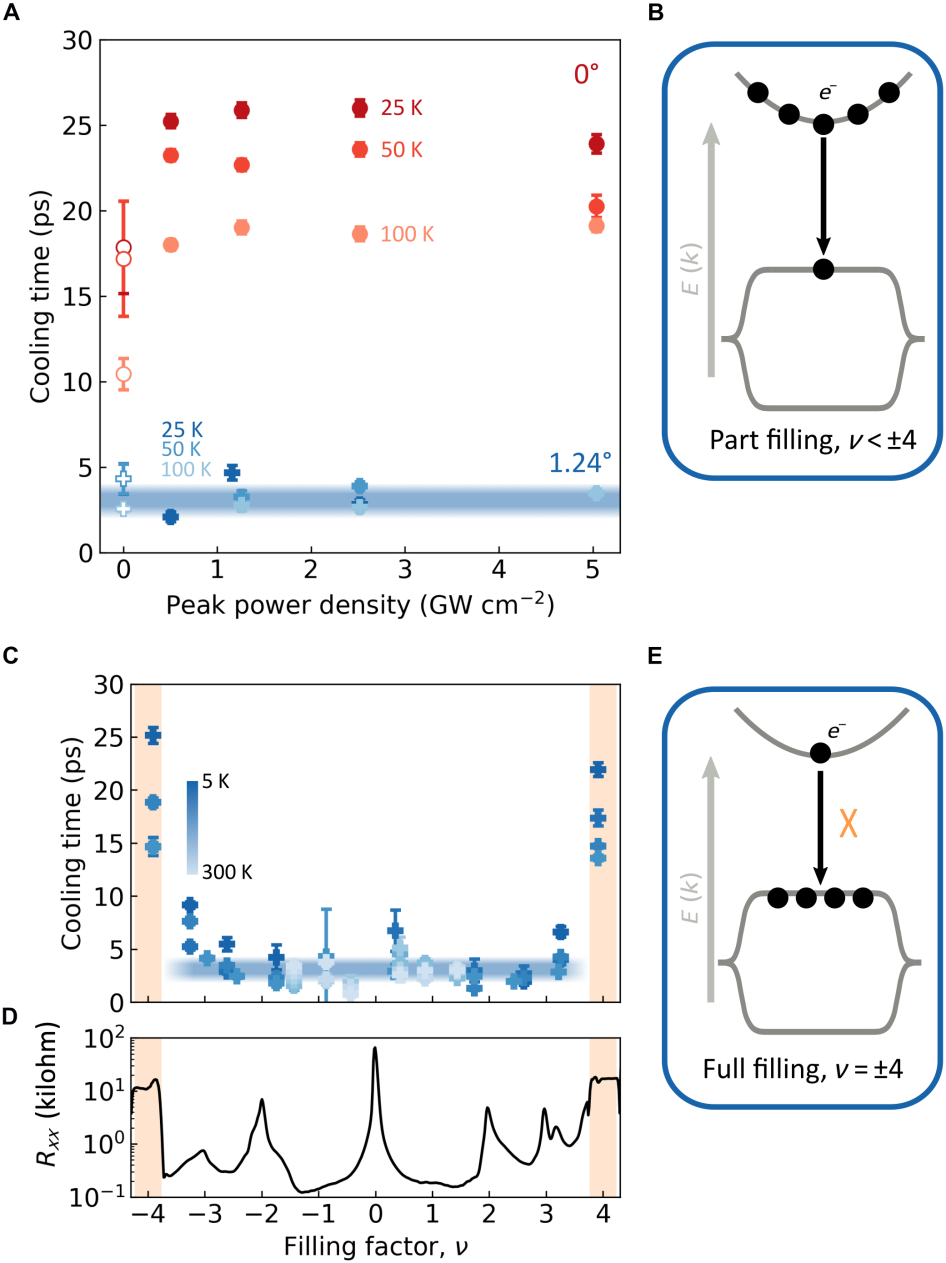
Origin of enhanced cooling in MATBG. (**A**) Dependence of cooling time on peak power density for BLG (red circles) and MATBG (blue pluses). The filled (open) shapes are measured using the TrPV (CW-PM) technique. The error bars signify the one sigma confidence interval from the fitting algorithm. (**B** and **E**) Schematics of cooling power in MATBG for part filling (B) and full filling (E) of the flat bands. For part filling, the interband transition is not rate-limiting as evidenced by the absence of a power dependence in (A). At full filling, cooling times are longer because of the interband bottleneck effect illustrated in (E). (**C** and **D**) Gate dependence of cooling time (C) and four-terminal resistance acquired at *T* = 35 mK (*R_xx_*) (D). Orange-shaded region highlights full filling of the moiré unit cell, where *R_xx_* and cooling time increase. In (A) and (C), the error bars signify the one sigma confidence interval from the fitting algorithm, and the thick blue line represents the cooling time obtained from the low-temperature model of Umklapp-assisted cooling (see main text).

We have ruled out the occurrence of diffusive cooling in MATBG because we observe cooling that is independent of spot size. Our observation that cooling in MATBG is independent of lattice temperature rules out cooling via intrinsic graphene acoustic and optical phonons, via disorder-assisted acoustic phonons, or via hBN substrate phonons. This is only true if the dynamics are not dominated by the electron temperature. To verify this, we study the effect of changing the laser power and therefore initial electron temperature (see Fig. 3A). This corresponds to increasing the population of the dispersive band (Fig. 3B). The peak power density is roughly five orders of magnitude larger for our pulsed laser experiment (TrPV) than our continuous-wave experiment (CW-PM). For the nontwisted BLG device, we observe somewhat slower cooling at higher incident powers, which has also been observed for high-quality monolayer graphene samples and was ascribed to a bottleneck involving optical and acoustic phonons (*39*). The role of electron temperature is minor for the relaxation dynamics of MATBG, suggesting that there are no electron-phonon or phonon-phonon bottlenecks. Our continuous-wave measurements with low peak power and therefore an electron temperature that is close to the lattice temperature also show fast cooling in MATBG across all temperatures. This confirms that the observed dynamics are not dominated by the electron temperature.

The much faster cooling for MATBG compared to BLG at low temperatures thus suggests that a completely different mechanism is responsible for cooling, outcompeting all currently known cooling mechanisms in nontwisted graphene: cooling via intrinsic graphene optical and acoustic phonons, via disorder, via substrate phonons, or via heat diffusion. We therefore explore the effect of the superlattice on electron cooling by examining the cooling time in MATBG as a function of filling factor (*v*) that represents the electronic occupation of the superlattice unit cell. For most filling factors (∣*v*∣ < 4), we observe a nearly-constant cooling time of 3 ps across a wide temperature range (5 to 300 K). However, at *v* = ±4, the cooling time increases markedly. Low-temperature transport measurements on the same device reveal an increase in resistance at the same voltages (see Fig. 3D), which confirms the full filling of the superlattice unit cell. We attribute this slower cooling at full filling to Pauli blocking of transitions from the dispersive band to the flat band, as illustrated in Fig. 3E. In fig. S10, we show that the cooling time increases strongly upon increasing laser power at full filling for the second MATBG device (θ = 1.06°). The strong dependence of cooling upon the flat band filling—with the cooling rates high at partial filling and lower at full filling—indicates that the moiré pattern and its low-energy phonons are crucial for explaining the ultrafast cooling dynamics observed in MATBG.

On the basis of the experimental observations, we infer that Umklapp scattering dominates the electron-phonon interaction in MATBG and is activated because of the presence of compressed Wannier orbitals at AA sites in the moiré lattice. This couples electrons to large-momentum phonons that would otherwise be forbidden by momentum conservation. To gain insight into the different mechanisms that govern electron-lattice cooling pathways in MATBG, we consider in detail the microscopic electron-phonon scattering processes. To this end, we consider a four-band model consisting of two nearly flat and two dispersive bands (Fig. 1D). There are two main types of electron-phonon scattering in this model, interband and intraband. The intraband processes for the intradispersive band and intraflat band transitions are different and must be evaluated separately. At temperatures higher than the bandgap, which corresponds to the highest temperatures in our measurement, the electrons are thermally excited to the dispersive bands allowing both dispersive and flat bands to contribute to cooling. To the contrary, when the electron temperature is low, all carriers reside in the flat band. Therefore, we consider two regimes: (i) the high-temperature regime (*T* ~ 150 to 300 K), where the dispersive bands contribute to the cooling process, and (ii) the low-temperature regime (*T* ∼ 10 K), wherein cooling is dominated by intraflat band processes. In both cases, we consider both the Umklapp and normal scattering contributions, finding that at the temperatures of interest (*T* > 10 K), electron-phonon Umklapp scattering consistently wins over normal scattering.

**Fig. 4.**
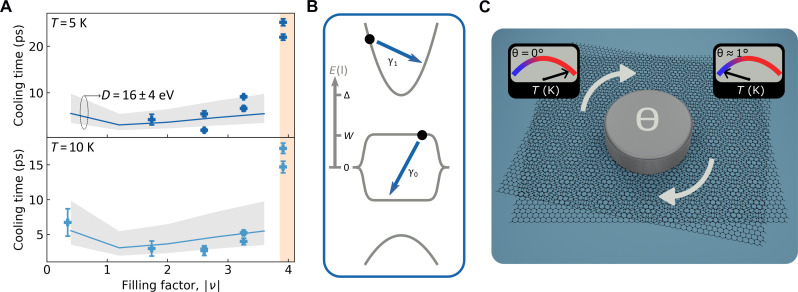
Quantitative comparison with Umklapp-assisted cooling. (**A**) Comparison between calculated (solid line) and experimental (symbols) cooling times for MATBG at 5 and 10 K (top and bottom). The gray-shaded region allows for uncertainty in the value of the deformation potential (*D* = 16 ± 4 eV). The error bars signify the one sigma confidence interval from the fitting algorithm. (**B**) Schematic of the model used for the calculations with two dispersive and two flat bands separated by an energy gap (Δ − *W*). γ_1_ and γ_0_ represent intradispersive band and intraflat band scattering processes, respectively. The low-temperature calculations shown in (A) consider only γ_0_. (**C**) Illustration showing the control of energy relaxation with twist angle.

For the first regime (high temperatures), we consider a four-band model consisting of two flat bands of bandwidth *W* and two dispersive bands with the eigenstate energies ε > Δ and ε < Δ (Δ > *W*) (see Fig. 4B). The dispersive bands are separated from the flat bands by a gap Δ − *W* (see Materials and Methods for details). A direct analysis based on Boltzmann theory yields cooling rates dominated by the intraband processes in the dispersive bands, whereas the interband processes have a minor contribution. Accounting for the electron-phonon Umklapp processes, we estimate the cooling rate as $\tau^{-1}=\frac{6\rho_{1}}{\pi T_{\text{el}}}\sum_{m}\left({\left\|g_{m}^{1,1}\right\|}^{2}+{\left\|g_{m}^{-1,-1}\right\|}^{2}\right)\omega_{m}^{2}$, where ρ_1_ is the DOS of the dispersive particle and hole bands labeled by *n* = ±1, *T*_el_ is the electron temperature, $g_{m}^{n,n}$ is the electron-phonon coupling constant in the *n*th band, and ω*_m_* is the phonon energy in the *m*th phonon band. Direct calculation gives cooling rates that are independent of the lattice temperature *T*_ph_, in agreement with the observed dynamics (see Fig. 2A).

For the regime of low temperatures, we describe the system using a model of a flat band with electron and hole subbands (see Materials and Methods for a detailed description of the model). For a quantitative comparison with the experimental results shown in Fig. 4A, we calculate the cooling power *J* accounting for the Umklapp processes assuming the Wannier function radius ξ = *a*/6, where *a* is the lattice parameter for the moiré structure (see Fig. 1C) (*28*). The cooling rate τ^−1^ is estimated from the calculated cooling power and specific heat using τ^−1^ = *J*/*C*(*T*_el_ − *T*_ph_); here, we calculate the specific heat *C* using the fluctuation formula, Eq. 2 in Materials and Methods. In Eq. 2, the temperature values are not constrained by the flat-band width and can be as large as the bandgap. The filling dependence of the cooling rate is shown in Fig. 4A. The calculated Umklapp-assisted cooling times as a function of the filling factor are in agreement with the experimental results. For the calculated cooling times, we used a deformation potential of 16 eV. This is close to the values reported for single-layer graphene (10 to 30 eV) (*33*, *57*–*59*). We therefore conclude that the Umklapp-assisted carrier cooling model, which has no freely adjustable parameters, reproduces the main experimental findings.

We note that the mechanism of Umklapp-assisted electron-phonon cooling is distinct from the previously identified disorder-assisted cooling mechanism (*31*). This disorder-assisted process occurs in graphene with relatively low charge mobility (high disorder) and speeds up electron-acoustic-phonon cooling by relieving the bottleneck due to limited phase space as a result of the small Fermi surface. The situation in MATBG differs from that of graphene in two ways. First, as the superlattice provides additional momentum recoil, MATBG does not require defects and/or disorder for electron-lattice cooling. Second, the formation of highly localized Wannier orbitals at AA sites in the moiré pattern modulates the electron-phonon interaction. These effects produce strong coupling of the electrons to moiré phonons even in the absence of disorder (*28*). Thus, Umklapp-assisted electron-phonon cooling enables the rare combination of high carrier mobility and ultrafast cooling dynamics, which persists from room temperature down to 5 K.

## DISCUSSION

Using two complementary optoelectronic techniques, we have demonstrated that hot carrier relaxation in twisted bilayer graphene is remarkably fast, with cooling times around 3 ps from room temperature down to 5 K. Our experiments based on a variation of twist angle, lattice temperature, spot size, electron temperature, and filling factor show that no conventional cooling mechanisms—based on optical phonons, acoustic phonons, substrates phonons, or thermal diffusion—can explain the experimental observations. Our Boltzmann theory calculations reveal that the origin of this ultrafast cooling in MATBG is electron-phonon Umklapp scattering—a fundamental scattering mechanism that overcomes the electron-phonon momentum mismatch. Crucial to the emergence of electron-phonon Umklapp scattering is the small superlattice Brillouin zone, spatially compressed Wannier orbitals, and low-energy moiré phonons. Hence, our results introduce twist angle as a route to control energy relaxation pathways.

Cooling measurements are predominantly sensitive to electron-phonon interactions and are less sensitive to electron-electron interactions. This presents a unique window of opportunity for probing underlying physics and an advantage compared to other measurement types that do not easily separate these two interactions. The finding that electron-phonon Umklapp scattering dominates ultrafast electron-phonon cooling is likely to have important implications for MATBG physics. Electron-phonon scattering plays an important role in charge transport, limiting the carrier mobility at high temperatures. This interaction also mediates the pairing interaction in Bardeen-Cooper-Schrieffer superconductors. Understanding the electron-phonon coupling could give important insights into the origin of superconductivity in MATBG (*25*, *60*), with direct implications for superconducting qubits and circuits (*13*–*15*). For metals, electron-electron Umklapp scattering gives rise to finite electrical resistance at low temperatures. In graphene/hBN superlattices and MATBG, this effect dominates transport at temperatures up to 10 K or higher, leading to excess resistivity and degradation of charge carrier mobility (*50*, *54*, *61*). In MATBG, electron-phonon Umklapp scattering could explain some of the open questions from electrical transport measurements, such as the strange metal phase or the role of phonons in superconductivity (*25*, *60*).

Electron-phonon Umklapp scattering remains efficient close to equilibrium as cooling is equally fast at low heating power. This is relevant for transport measurements and low-power or energy-harvesting applications (*16*). Last, the short hot carrier lifetime, as a result of Umklapp-assisted electron-phonon cooling, will enable the development of ultrafast photodetectors based on moiré materials for sensing applications in the visible, infrared, and terahertz spectral ranges particularly in cases where fast switching rates and low operating temperatures are required, such as in space and quantum applications.

## MATERIALS AND METHODS

### Device fabrication

The MATBG devices were fabricated using a cut and stack technique. All flakes were first exfoliated on a Si/SiO_2_ (285 nm) substrate and later picked up using a polycarbonate (PC)/polydimethylsiloxane stamp. All the layers were picked up at a temperature of ~100°C. We used an atomic force microscopy tip to cut the graphene to avoid strain during the pickup process. The PC/polydimethylsiloxane stamp picks up first the top graphite layer, the top hBN and the first graphene layer. Before picking up the second graphene layer, we rotate the stage by an angle of 1.1° to 1.2°. Last, the stamp picks up the bottom hBN and bottom graphite gates. We drop the finalized stack on a Si/SiO_2_ substrate by melting the PC at 180°C (see fig. S1A). The resulting stack is etched into a Hall bar using a CHF_3_/O_2_ plasma, and a one-dimensional contact is formed by evaporating Cr (5 nm)/Au (50 nm) (see fig. S1B). We etch a narrow channel of ~150 nm in the top gate using an O_2_ plasma. Before etching the top gate, the device was characterized at *T* = 35 mK to identify the pair of contacts closest to the magic angle (θ ~ 1.1°). The junction was made in between this pair of contacts.

We fabricated three different devices using this fabrication method: Device 1, with a twist angle of 1.24° ± 0.02°, comprises top and bottom graphite gates, two encapsulating hBN layers, and the twisted bilayer graphene. The thickness of both graphite gates is *d* = 1.7 ± 0.7 nm, while the top and bottom hBNs have respective thicknesses of *d* = 30 ± 3 nm and *d* = 20 ± 3 nm. Device 2, with a twist angle of 0°, has a single top graphite gate of thickness *d* = 1.3 ± 0.7 nm, two encapsulating hBN layers, and the bilayer graphene. The thicknesses of top and bottom hBN are *d* = 20 ± 3 nm and *d* = 18 ± 3 nm, respectively. Device 3, with a twist angle of 1.06° ± 0.02°, features top and bottom graphite gates, twisted bilayer graphene, and two encapsulating hBN layers. Thicknesses for top and bottom gates are *d* = 2 ± 0.7 nm and *d* = 2.3 ± 0.7 nm. The thickness of top and bottom hBN is *d* = 8 ± 3 nm and *d* = 14 ± 3 nm, respectively. Using the transfer matrix method, we calculated the absorption in the bilayers of these three samples, accounting for reflections at the different material interfaces, and found absorptions at 1030 nm (1550 nm) of 1.7% (4.20%), 2.04% (5.22%), and 1.94% (4.60%) for devices 1, 2, and 3, respectively (see fig. S11).

### Twist angle extraction

The twist angle θ is extracted from the superlattice carrier density of the full band *n*_s_ by applying the relation $n_{s}=8\theta^{2}/\sqrt{3}a^{2}$, where *a* = 0.246 nm is the graphene lattice constant. First, we calibrate the gate-induced carrier density using the Hall effect data at ±1*T*. In the carrier density region close to charge neutrality, the Hall carrier density *n*_H_ = −*B*/*eR_xy_* should closely follow the gate induced carrier density *n*_H_ = *n* (see fig. S2). By plotting *n*_H_ versus *V*_g_ and fitting this slope around charge neutrality, we can obtain the capacitance of the device and therefore extract the real carrier density *n*. Then, we extract the carrier density corresponding to a fully filled superlattice unit cell; in this case, we find it to be *n*_s_ = (3.58 ± 0.10) × 10^12^ cm^−2^. Last, using the above relation, we extract a twist angle θ = 1.24° ± 0.02°. In Supplementary Text and fig. S3, we verify that there is minimal twist angle disorder in the junction region.

### Transport measurements

Low-temperature transport measurements were carried out in a dilution refrigerator (Bluefors SD250) with a base temperature of 20 mK. Standard low-frequency lock-in techniques (Stanford Research SR860 amplifiers) were used to measure *R_xx_* with an excitation current of 10 nA at a frequency of 13.11 Hz.

### Optoelectronic measurements

In TrPV experiments, which were performed using pulsed light with a wavelength of 1030 nm, we vary the delay time (*dt*) between the arrival of two ultrafast pulses (*29*, *46*, *47*). Because of the nonlinear relationship between carrier temperature and optical heating, we observe a dip in the photovoltage when the two pulses arrive at the same time (*dt* = 0) (see Fig. 1C and figs. S6 and S7). At longer delay times, the signal recovers to its maximal value. We obtain the cooling time by describing the observed dynamics with an exponential function. For heterodyne photomixing (CW-PM) experiments, the wavelength detuning between the two continuous-wave lasers, each with a wavelength around 1550 nm, creates an optical beating (*48*, *49*). The photovoltage oscillates at the beating frequency. Because of the competition between beat frequency (Ω) and the characteristic cooling time (τ_e_), we observe a peak for Ω = 0, whereas the oscillations are damped when Ω^−1^ ≪ τ_e_ (see figs. S8 and S9). The frequency response takes the form of a Lorentzian function of width Γ, from which we extract the cooling time as Γ = 1/πτ_e_ (*48*).

### Cooling rate at low temperatures

The cooling rate in Fig. 3F is estimated by $\frac{J\left(T_{\text{el}},T_{\text{ph}}\right)}{C\left(T_{\text{el}}\right)\left(T_{\text{el}}-T_{\text{ph}}\right)}$, where *J* is the cooling power, *C*(*T*_el_) is the electron specific heat, and *T*_el_ (*T*_ph_) is the electron (phonon) temperature. To evaluate *J* and *C*, we consider an effective two-band model similar to pristine graphene used in (*28*). Following the previous study, we use the electron-phonon interaction for the Wannier orbital radius ξ = *a*/6, where *a* is the lattice parameter. In the Boltzmann theory, the cooling power *J* by electron-phonon scattering reads (*28*)

J=∑n,n′Jn,n′,Jn,n′=2πV2∑m,k→,k→′‖gk→−k→′¯,mnn′‖2ωk→−k→′¯,m2Nk→−k→′¯,m×fk→′n′[1−fk→n]eβphωk→−k→′¯,m−fk→n[1−fk→′n′]×δ(εk→′n′−εkn−ωk→−k→′¯,m) (1)

where *J*_*n*,*n*′_ is the contribution from the scattering between *n*th and *n*th bands, *V* is the volume of the system, $g_{\bar{\vec{k}-\vec{k}\prime },m}^{nn\prime }$ is the coupling constant, $\varepsilon_{\vec{k}n}$ is the one-particle eigenenergy of the eigenstate in *n*th band with momentum $\vec{k}$, $\omega_{\vec{q}m}$ is the phonon eigenenergy in the *m*th band with momentum $\vec{q}$, and β_el_ = 1/*k*_B_*T*_el_ (β_ph_ = 1/*k*_B_*T*_ph_) is the inverse temperature of electrons (phonons) with *k*_B_ being the Boltzmann constant; $f_{\vec{k}n}=\frac{1}{e^{\beta_{el}\left(\varepsilon_{\vec{k}n}-\mu \right)}+1}$ and $N_{\vec{q}m}=\frac{1}{e^{\beta_{\text{ph}}\omega_{\vec{q}m-1}}}$ are the Fermi and Bose distribution functions, respectively. The estimation of specific heat uses the fluctuation formula

$$C\left(T\right)=k_{B}\left[\langle \varepsilon_{n\vec{k}}^{2}\rangle -\frac{{\langle \varepsilon_{n\vec{k}}^{2}\rangle }^{2}}{\langle 1\rangle }\right]$$ (2)

$$\left\langle O\right\rangle =\sum_{n}\int \frac{{\mathrm{dk}}^{d}}{{\left(2\pi \right)}^{d}}\frac{\beta^{2}O_{n\vec{k}}}{4{\text{cosh}}^{2}\left[\frac{\beta \left(\varepsilon_{n\vec{k}}-\mu \right)}{2}\right]}$$ (3)

Note that the common formula for Fermi-degenerate electron systems does not apply here as the temperature exceeds the Fermi energy at *T* ≳ 100 K. This model gives a good approximation when the temperature is much lower than the energy gap separating the flat band from high-energy dispersive bands.

### Cooling rate at high temperatures

At high temperatures, we cannot neglect the high-energy bands because the electron temperature exceeds the bandgap. In such a case, the Umklapp scattering involving high-energy phonons contributes to electron cooling due to a large number of high-energy phonons. Hence, we also expect that Umklapp scattering plays a key role in the high-temperature regime.

To study the electron-lattice cooling involving the interband processes, we assume the electrons only couple to phonons with energies below a cutoff Λ_ph_. This assumption is justifiable in a system where the electron-phonon coupling between the electrons and the acoustic phonons reduces exponentially as the momentum increases. In a system with compact Wannier orbitals, Λ_ph_ becomes a few times higher than the energy of folded acoustic bands. Hence, a large Λ_ph_, considerably larger than the phonon bandwidth of the folded acoustic phonons, represents the enhanced coupling by compact Wannier orbitals. Below, we label the folded acoustic bands by an integer *m* and define the high-temperature limit as *T*_el_ > *T*_ph_ ≫ Λ_ph_.

At high temperatures, the cooling power in Eq. 1 reads

$$\begin{array}{c} J_{nn\prime }=\frac{\pi }{V}\sum_{m}\left\|g_{m}^{nn\prime }\right\|\omega_{m}^{2}\rho_{n}\rho_{n}^{´}\left[T_{\text{el}}-T_{\text{ph}}\right]\times \\ \left\{\text{tanh}[\frac{\beta (b_{nn\prime }^{m}-\mu )}{2}]-\text{tanh}[\frac{\beta (a_{nn\prime }^{m}-\mu )}{2}]\right\} \end{array}$$

where ρ_*n *_is the DOS for the *n*th band (we assume a constant DOS with the bandwidth *W_n_*) and $a_{\mathrm{nn}\prime }^{m}=\text{max}(\varepsilon_{n}^{-}-\varepsilon_{n\prime }^{-}-\omega_{m})[b_{\mathrm{nn}\prime }^{m}=\text{min}(\varepsilon_{n}^{+}-\varepsilon_{n\prime }^{-}-\omega_{m})]$ with $\varepsilon_{n}^{\pm }$ being the energy of the top and bottom edge of the electron band. Here, we approximated the phonon energy as $\omega_{n\vec{k}}∼\omega_{n}$ considering the small Brillouin zone, and the coupling constant $g_{\bar{\vec{k}-\vec{k}\prime },m}^{nn\prime }∼g_{m}^{nn\prime }$ that is valid in the small orbital radius limit.

We apply the above formula to a four-band model consisting of two flat and two dispersive bands. The two flat bands are at energies 0 ≤ ε ≤ *W* and −*W* ≤ ε ≤ 0 with DOS ρ_0_, and the two dispersive bands are *W* < Δ ≤ ε ≤ Λ and −Λ ≤ ε ≤ −Δ < −*W* with DOS ρ_1_ (Fig. 4B). To the leading order in *T*_el_, the cooling power reads

$$J=\pi \sum_{m}\left({\left\|g_{m}^{1,1}\right\|}^{2}+{\left\|g_{m}^{-1,-1}\right\|}^{2}\right)\omega_{m}^{2}\left[T_{\text{el}}-T_{\text{ph}}\right]\rho_{1}^{2}$$

Hence, the cooling rate becomes $\tau^{-1}=\frac{6\rho_{1}}{\pi {\mathrm{VT}}_{\text{el}}}\sum_{m}({\left\|g_{m}^{1,1}\right\|}^{2}+{\left\|g_{m}^{-1,-1}\right\|}^{2})\omega_{m}^{2}$, independent of phonon temperature, *T*_ph_.

We note that we do not observe this dependence on electron temperature when increasing the incident power and therefore—supposedly—the initial *T*_el_. The reason is that efficient cooling via optical phonons results in ultrafast cooling to an electron temperature of ~300 K, below which cooling via optical phonons becomes inefficient, namely, slower than a picosecond (*39*). As a result, the cooling dynamics that we observe are those associated with the cooling from this initial *T*_el_, which is independent of incident power. This cooling channel via optical phonons is disregarded in our model of cooling via moiré phonons because, at temperatures of interest, the optical phonons are not expected to provide a dominant contribution.
