# Supplementary material for: Ultrafast Umklapp-assisted electron-phonon cooling in magic-angle twisted bilayer graphene
Source: Sci Adv. 2024 Feb 9;10(6):eadj1361. doi: 10.1126/sciadv.adj1361 (PMC10857426; doi:10.1126/sciadv.adj1361)
Supplement: Supplementary file 1 — Supplementary Text Figs. S1 to S11 [file sciadv.adj1361_sm.pdf]

Science Advances
AAAS

Supplementary Materials for

Ultrafast Umklapp-assisted electron-phonon cooling in magic-angle twisted bilayer graphene

Jake Dudley Mehew *et al.*

Corresponding author: Klaas-Jan Tielrooij, klaas.tielrooij@icn2.cat

Sci. Adv. **10**, eadj1361 (2024)
DOI: 10.1126/sciadv.adj1361

This PDF file includes:

Supplementary Text
Figs. S1 to S11

SUPPLEMENTARY INFORMATION

SUPPLEMENTARY TEXT

Estimating cooling times in untwisted graphene

The hot electron cooling time for energy transfer to acoustic phonons in monolayer graphene is given by $\tau_{AP} \approx 848/(D^2 T_L^2)$ [μs] (*30*), where D is the deformation potential in eV. This expression is valid in the neutral limit ($T_F < T_e$) and close to equilibrium ($T_e \gtrsim T_L$). $T_{e/L/F}$ is the electron/lattice/Fermi temperature (*30*). Taking $D = 20$ eV, we calculate a cooling time of $\tau_{AP} = 450$ ns for $T_L = 25$ K.

In disorder-assisted or supercollision cooling (*31*, *33*, *35*), the dependence on lattice temperature is given by:

$$\tau_{SC} = \frac{\alpha}{3AT_L}, \text{ with}$$
$$\alpha = \frac{2\pi E_F k_B^2}{3\hbar^2 v_F^2} \text{ and } A = 9.62\frac{g^2\nu^2(E_F)k_B^3}{\hbar k_F \ell}.$$

Here, g is the electron-phonon coupling, $\nu(E_F)$ is the density of states at the Fermi level per valley/spin flavour, k_F is the Fermi wavevector and ℓ is the mean free path. In high-quality samples and at cryogenic temperatures, the device size typically limits the latter. For low doping levels (10^{12} cm^{-2}), $0.1 < \ell < 2$ μm and $T = 25$ K, $\tau_{SC} = 0.5 - 11$ ns.

Cooling due to lateral diffusion

The lateral diffusion of photoexcited carriers reduces the hot electron temperature when the cooling length is greater than the laser spot size. This effect is particularly relevant in high-mobility samples, as the Wiedemann-Franz law relates electrical to thermal conductivity (*38*). At low lattice temperatures efficient heat conduction manifests in our experiments as a shorter cooling time. By considering the spatial evolution of a Gaussian heat spot induced by the laser pulse (*39*), we describe the temperature dynamics by:

$$T_e(t) = 2\pi A_{pu} A_{pr} \frac{\sigma_{pu}^2 \sigma_{pr}^2}{\sigma_{pu}^2 + \sigma_{pr}^2 + 2Dt},$$

where A and σ are the peak intensity of the pump (pu) and probe (pr). Clearly, this effect is greater for smaller spot sizes and larger electronic heat diffusivities (D). Using a diffusivity

of $D = 750$ cm^{2}s^{-1}, and pump-probe spot sizes of $\sigma \approx 0.9$ μm we find a cooling time of $\tau_{diff} \approx 18$ ps. For $\sigma \approx 1.4$ μm, $\tau_{diff} \approx 45$ ps, see Fig. 2C.

Twist-angle disorder

In Supp. Fig. S3, we investigate the influence of twist angle disorder on the electrical transport at $T = 35$ mK. At the junction contact, the twist angle is $\theta = 1.24°$ and we observe sharp resistance peaks at $\nu = \pm 2$ arising from correlated insulating states. The contacts at the top of the junction display a shoulder around $\nu = -2$ that indicates a mixing of two angles ($\theta = 1.24 - 1.28°$). For the contacts at the bottom of the junction the angle is $\theta = 1.24°$. From this we conclude that there is minimal twist angle disorder in the proximity of the pn-junction.

SUPPLEMENTARY FIGURES

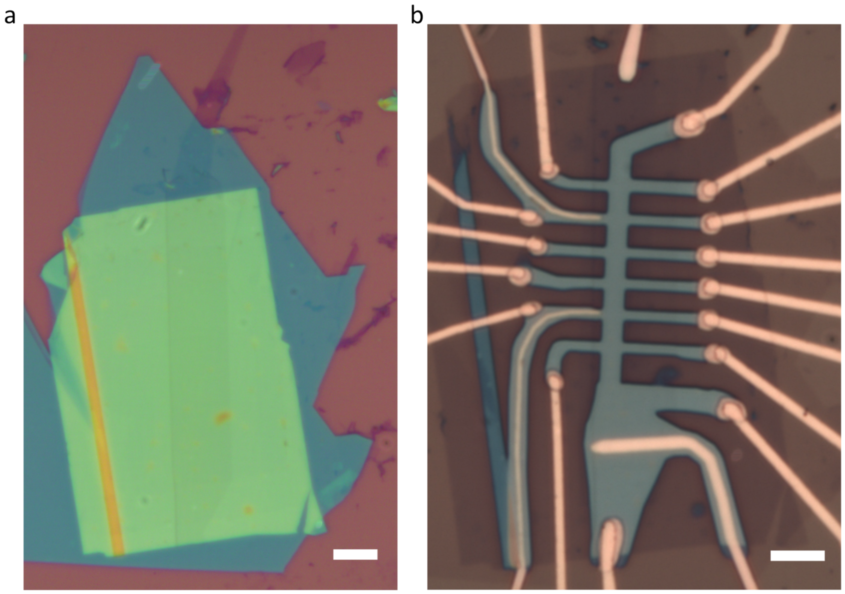

Fig. S1. **Optical images of the device before and after nanofabrication.** **a**, Heterostructure stack dropped on a Si/SiO_2 substrate. **b**, Finalised device after etching Hall bar and metallisation. Both scale bars are 5 μm.

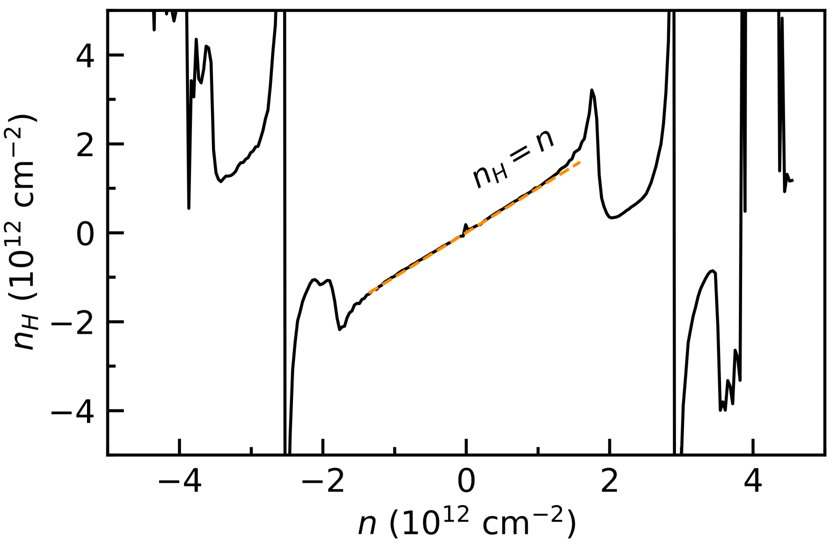

Fig. S2. **Low field Hall effect at 1.8 K.** Hall carrier density n_H vs n. In the region close to charge neutrality $n_H = n$, which allows us to calibrate the relationship between V_g and n to extract the twist angle.

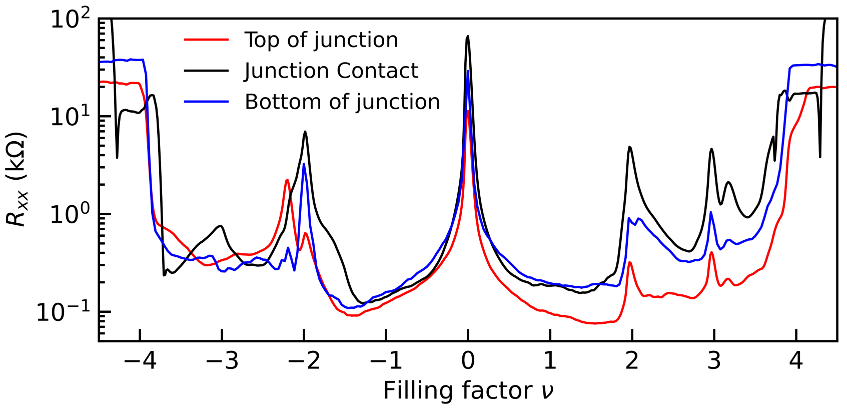

Fig. S3. **Twist-angle disorder.** Longitudinal resistance (R_{xx}) vs. filling factor (ν) for contacts around the junction.

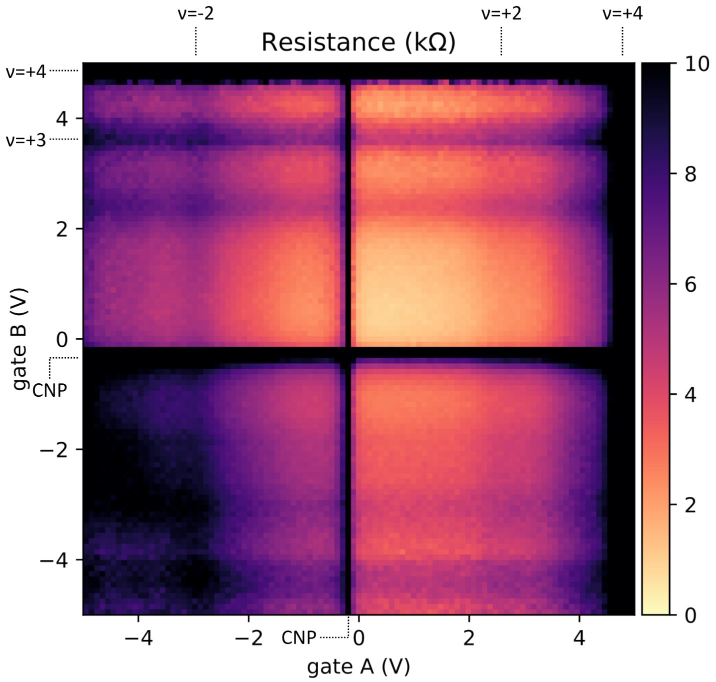

Fig. S4. **Gate-dependent resistance.** Dual gate map of the four-probe resistance of MATBG ($\theta = 1.24°$) at T=3.6 K. The maxima in resistance correspond to the charge neutrality points (CNPs) and integer filling factors ($\nu = \pm 2, \pm 3, \pm 4$).

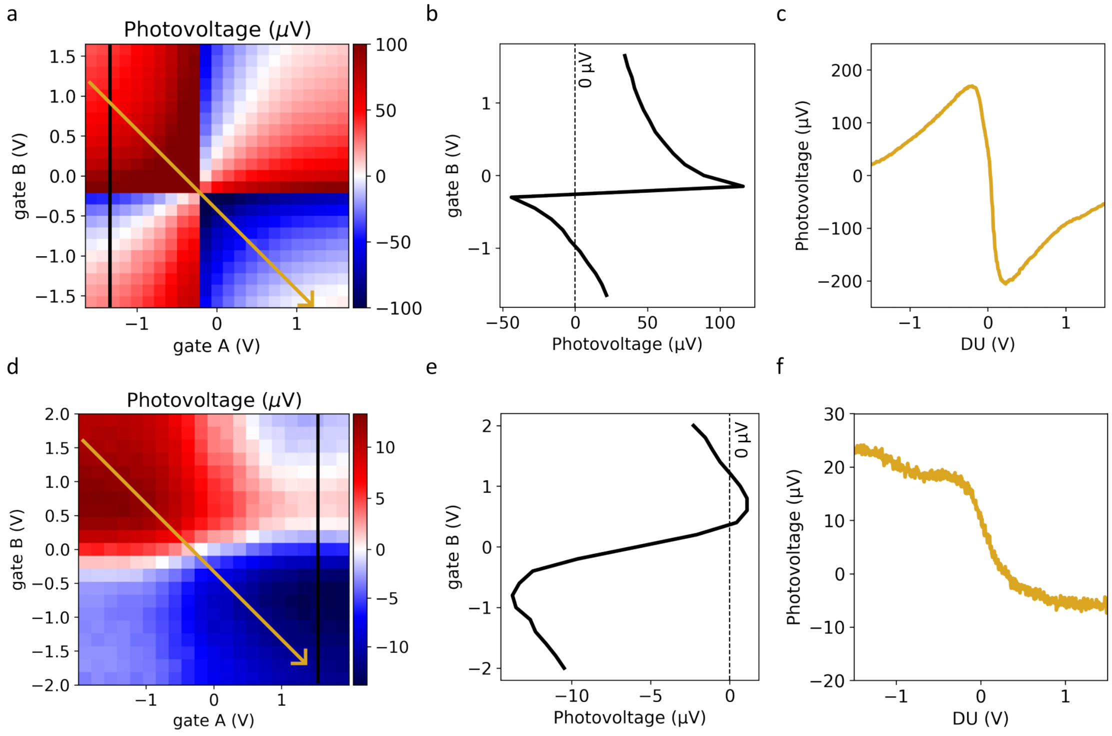

Fig. S5. **Photothermoelectric effect.** Gate dependent photovoltage for **a, b, c** MATBG ($\theta = 1.24°$, $T = 10$ K) and **d, e, f** BLG ($\theta = 0°$, $T = 200$ K). The dual gate photovoltage maps (**a, d**) reveal a six-fold symmetry for both samples. The photovoltage extracted at a fixed gate A voltage (solid black lines in **a, d**), crosses zero (dashed line) at two gate B voltages, **b, e**. This indicates that the origin of the photovoltage is the photothermoelectric effect for twisted and non-twisted bilayer graphene. Panels **c, f**, show the photovoltage obtained by applying opposite voltages (with respect to the Dirac points) to either side of the pn junction, indicated by the yellow arrow in **a, d**.

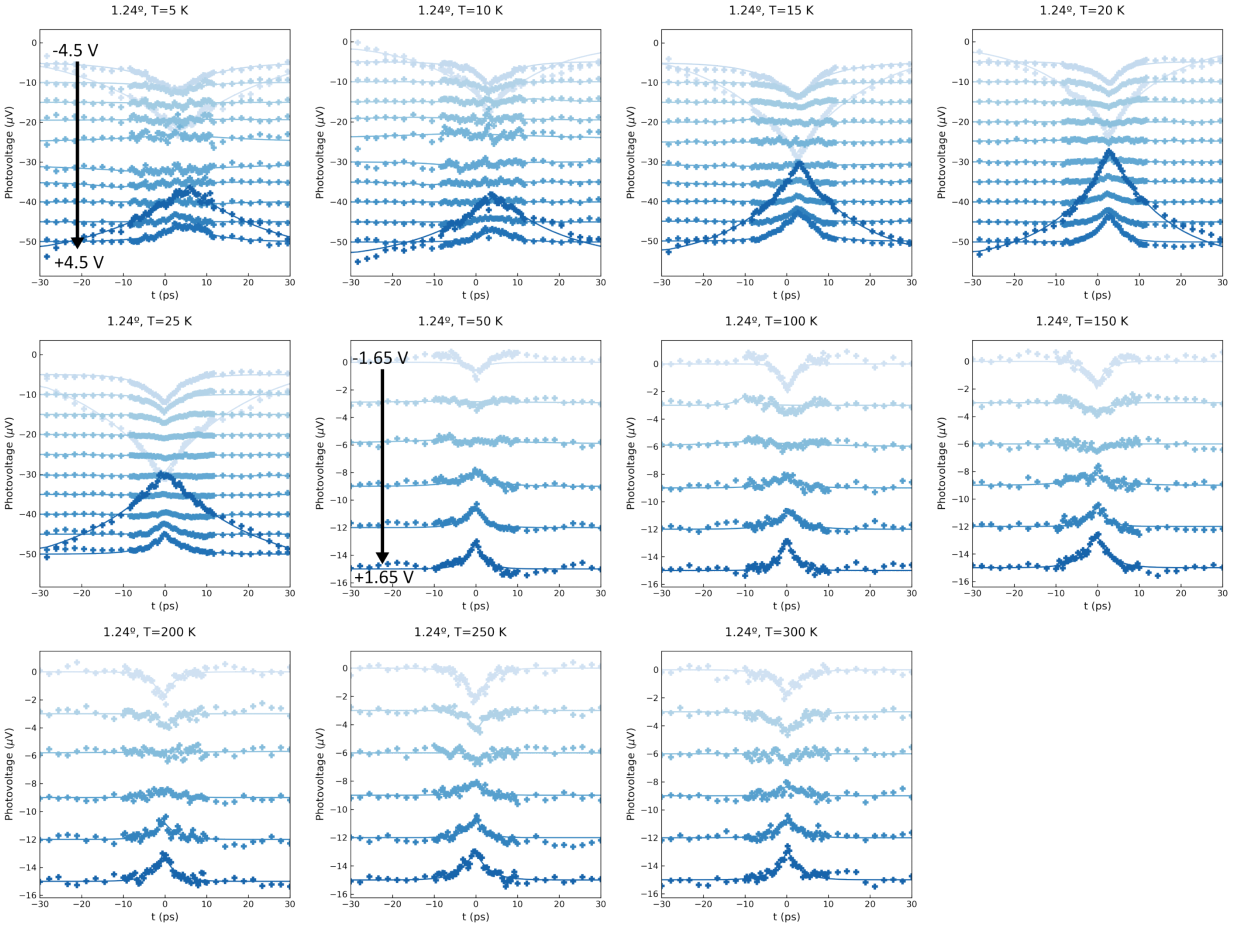

Fig. S6. TrPV dips for the MATBG ($\theta = 1.24°$) device as a function of DU vector (indicated by arrow) and temperature (see plot title). Each time trace has been offset for clarity.

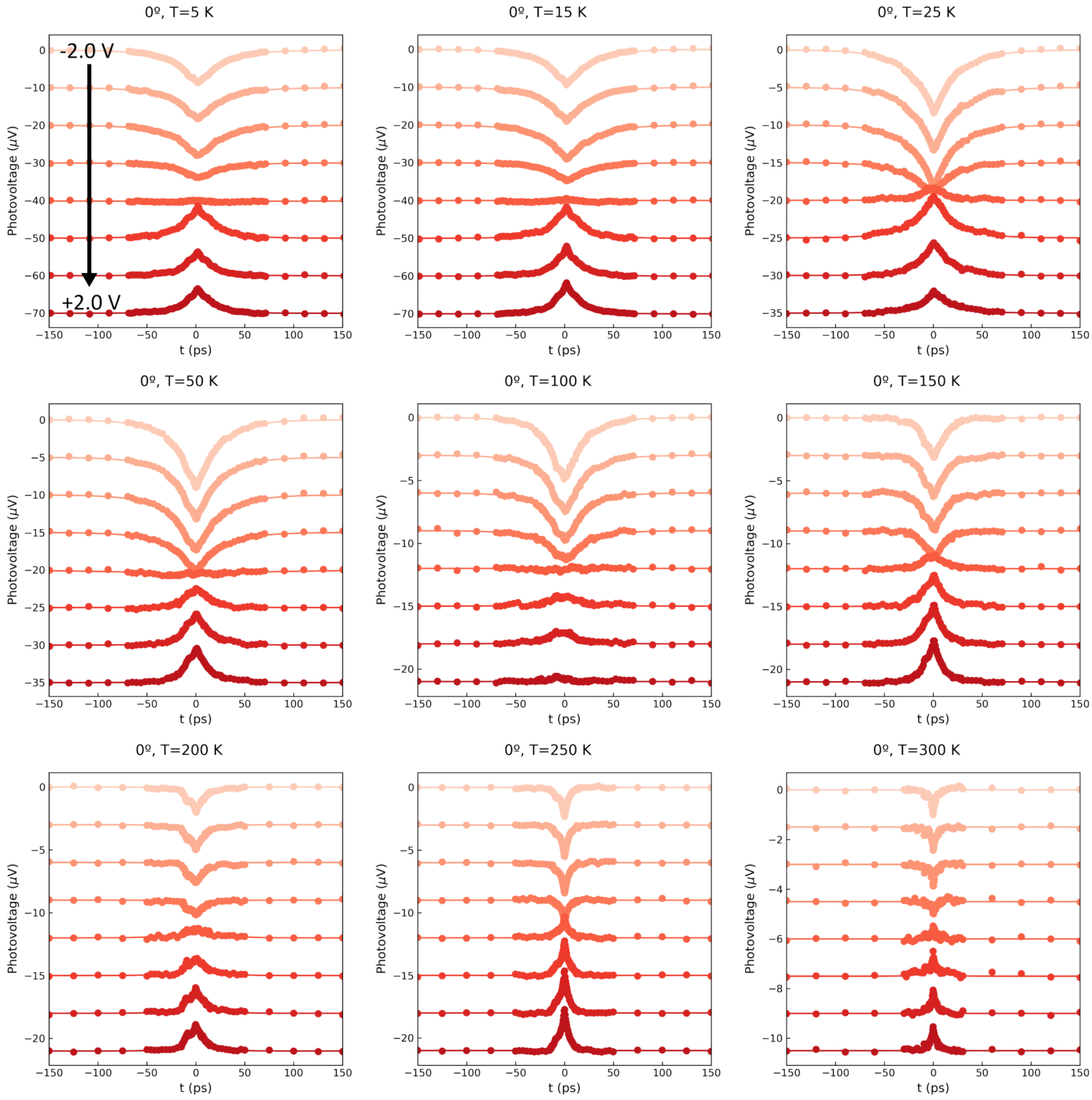

Fig. S7. TrPV dips for the BLG ($\theta = 0°$) device as a function of DU vector (indicated by arrow) and temperature (see plot title). Each time trace has been offset for clarity. The slower cooling at low temperatures produces a broader dip.

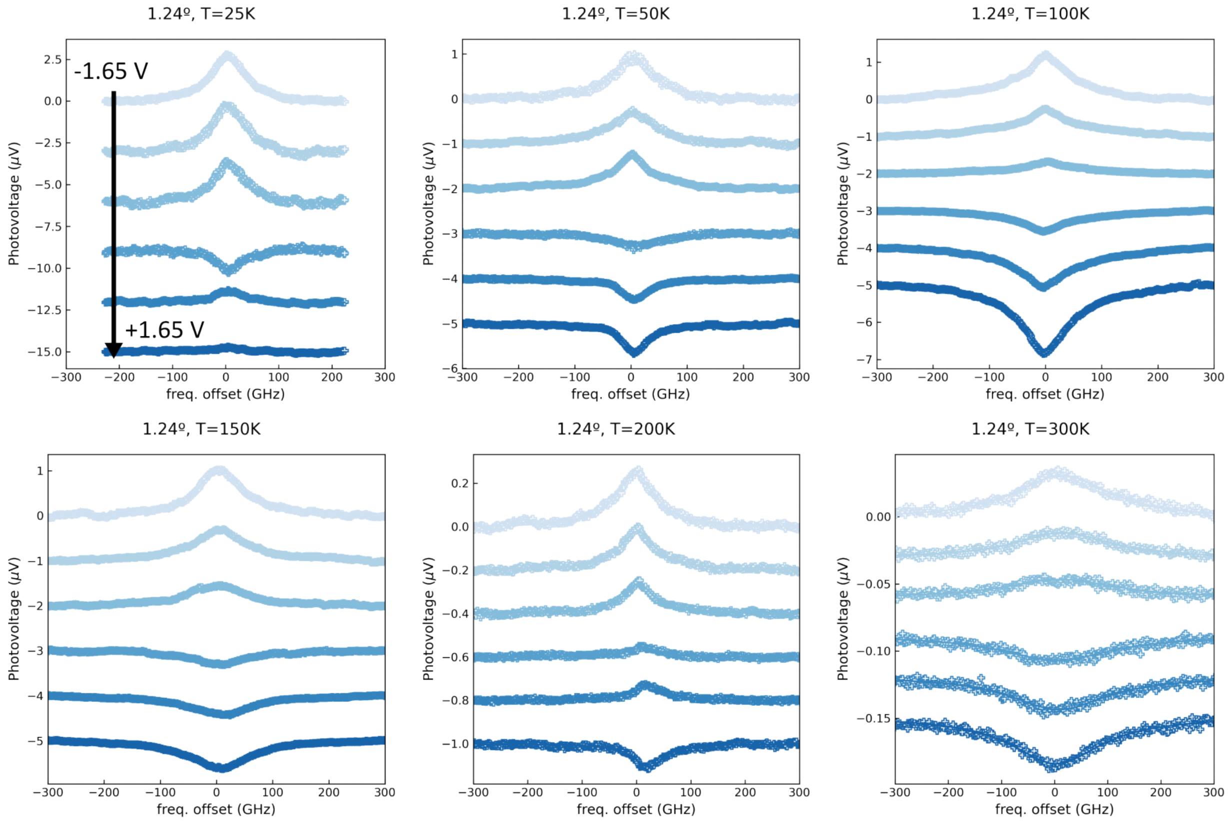

Fig. S8. CW-PM peaks for the MATBG ($\theta = 1.24°$) device as a function of DU vector (indicated by arrow) and temperature (see plot title). Each frequency sweep has been offset for clarity.

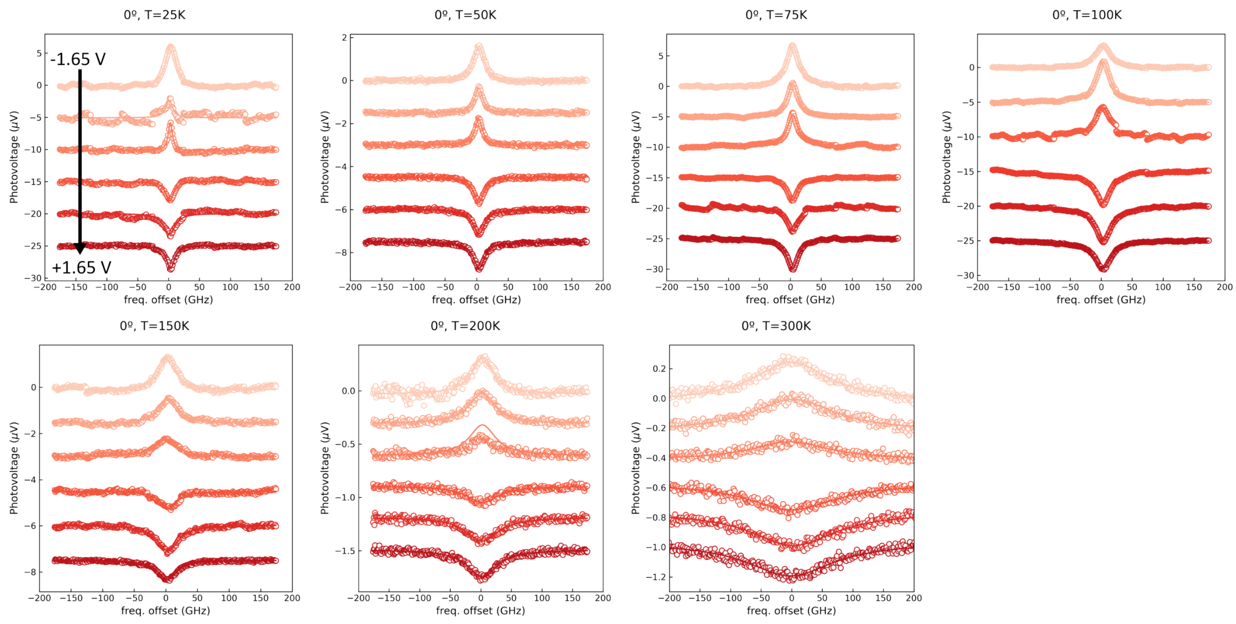

Fig. S9. CW-PM peaks for the BLG ($\theta = 0°$) device as a function of DU vector (indicated by arrow) and temperature (see plot title). Each frequency sweep has been offset for clarity. The slower cooling at low temperatures produces a narrower peak.

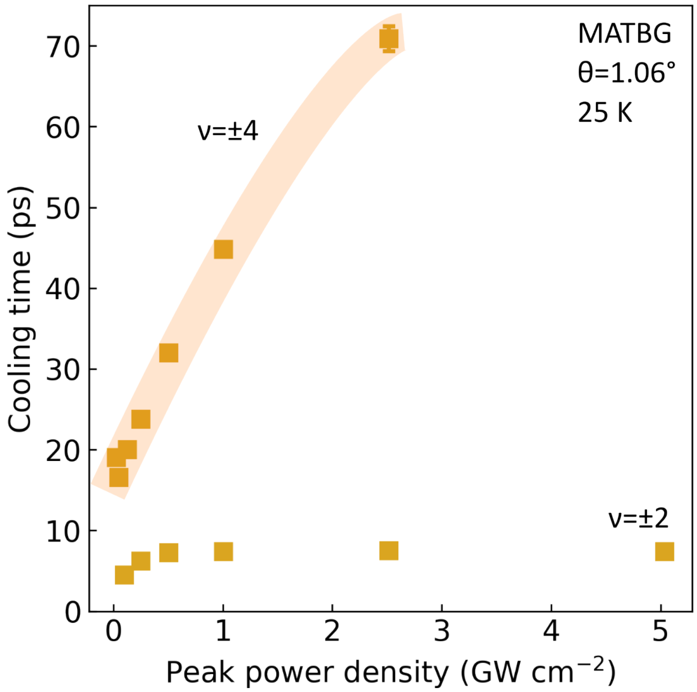

Fig. S10. Power dependence of cooling time for a second MATBG device ($\theta = 1.06°$) at 25 K. The electron relaxation bottleneck at full filling ($\nu = \pm 4$) leads to slower cooling time for higher laser powers. The orange line is a guide to eye.

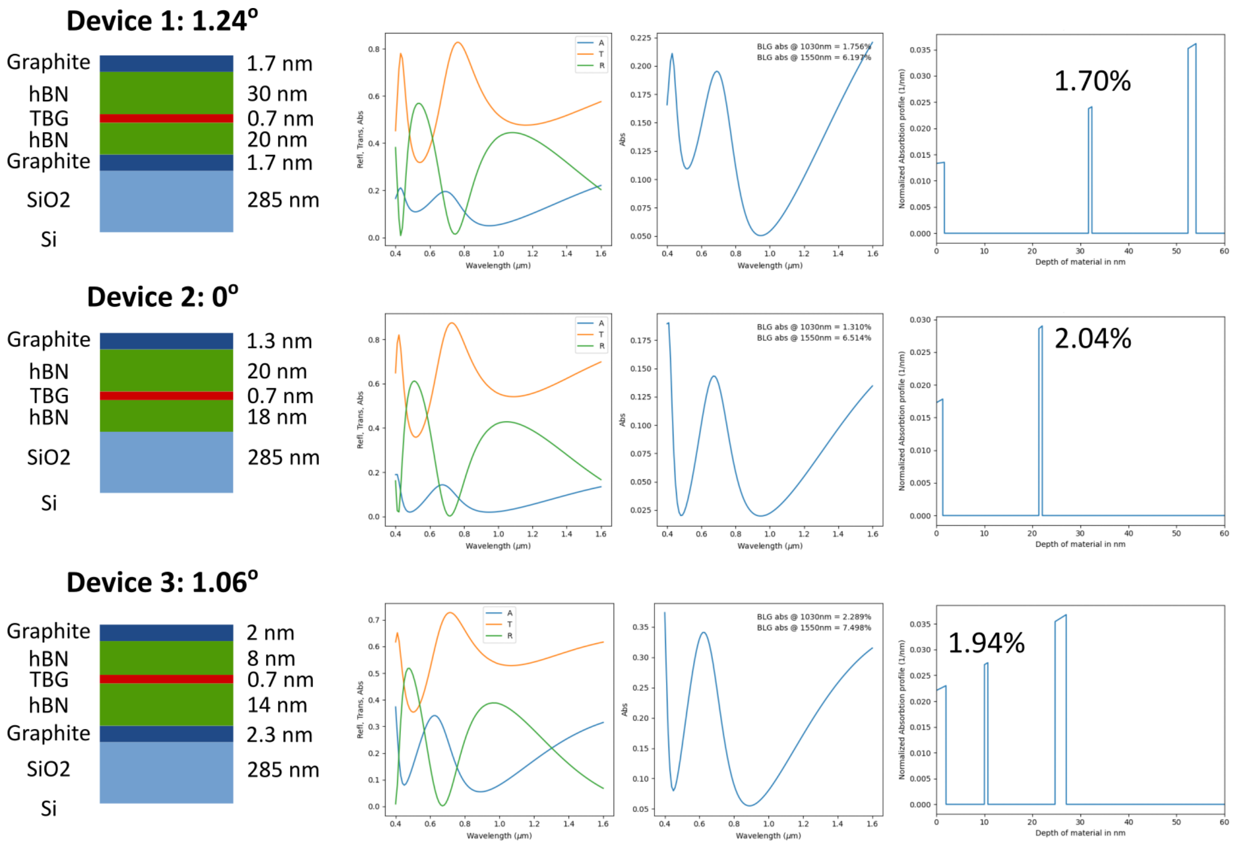

Fig. S11. Calculation of the absorption in the bilayer region of each of the three devices, based on the transfer matrix method. This is for an incident wavelength of 1030 nm.
